# Utilization of rare codon-rich markers for screening amino acid overproducers

**DOI:** 10.1038/s41467-018-05830-0

**Published:** 2018-09-06

**Authors:** Bo Zheng, Xiaoyan Ma, Ning Wang, Tingting Ding, Liwei Guo, Xiaorong Zhang, Yu Yang, Chun Li, Yi-Xin Huo

**Affiliations:** 1 School of Life Science, Beijing Institute of Technology, No. 5 South Zhongguancun Street, 100081 Beijing, China; 2 UCLA Institute of Advancement (Suzhou), 10 Yueliangwan Road, Suzhou Industrial Park, 215123 Suzhou, China; 3 Institute of Biophysics, Chinese Academy of Sciences, 15 Datun Road, Chaoyang District, 100101 Beijing, China; 4 School of Chemistry and Chemical Engineering, Beijing Institute of Technology, No. 5 South Zhongguancun Street, 100081 Beijing, China

## Abstract

The translation of rare codons relies on their corresponding rare tRNAs, which could not be fully charged under amino acid starvation. Theoretically, disrupted or retarded translation caused by the lack of charged rare tRNAs can be partially restored by feeding or intracellular synthesis of the corresponding amino acids. Inspired by this assumption, we develop a screening or selection system for obtaining overproducers of a target amino acid by replacing its common codons with the corresponding synonymous rare alternative in the coding sequence of selected reporter proteins or antibiotic-resistant markers. Results show that integration of rare codons can inhibit gene translations in a frequency-dependent manner. As a proof-of-concept, *Escherichia coli* strains overproducing l-leucine, l-arginine or l-serine are successfully selected from random mutation libraries. The system is also applied to *Corynebacterium glutamicum* to screen out l-arginine overproducers. This strategy sheds new light on obtaining and understanding amino acid overproduction strains.

## Abstract (web summary)

Current toxic analogues-based amino acid overproducer screening method cannot provide accuracy, sensitivity and high throughput simultaneously. Here, the authors use rare codon rich marker for the selection of overproducers of multiple amino acids from random mutation libraries of *E. coli* and *C. glutamicum*.

## Introduction

**Fig. 1:**
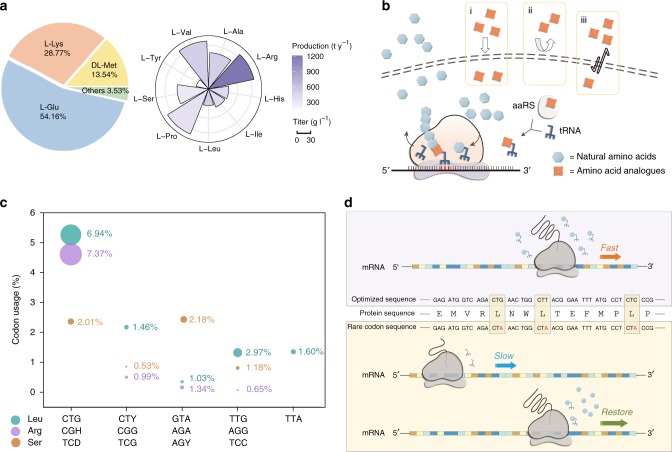
Amino acid productions and codon usage. **a** Global productions of amino acids (left), the annual productions (right, represented by color intensity), and the fermentation titer (right, represented by bar height) for nine selected amino acids. **b** After taken up by the cells (i), the amino acid analogues (orange square) compete with the corresponding natural amino acids (blue hexagon) for the finite tRNAs, a step catalyzed by the aminoacyl-tRNA synthetase (aaRS). The analogues could be blocked (ii) or pumped outside of the cells (iii). **c** Codon usage and the fraction of tRNAs (bubble diameter) in *E. coli*^22^. The fraction of individual tRNA out of the total tRNA was derived from *E. coli* W1485, a K strain derivative at a growth rate of 0.4 doublings h^–1^. **d** For an exogenous gene, replacing its codons (e.g. leucine codon) with synonymous ones that are recognized by the most abundant tRNAs for a specific host would typically improve the expression of the desired protein (upper box). On the contrary, the rare tRNAs have lower chances to be charged with the corresponding amino acids, switching to the rare alternatives (e.g. leucine codon CTA for *E. coli*) that pair with the low-abundance tRNAs would dramatically slow down protein expression (lower box). Theoretically, the retarded protein expression should be restored by increased intracellular concentrations of the corresponding amino acids

The amino acids have a multi-billion-dollar market with applications in food, animal feed, pharmaceutical, and cosmetic industries^1^. The worldwide market for amino acids represented overall $8.8 billion in 2007 and increased 3.47% per year^2^ to over $10 billion in 2015. Although large-scale microbial fermentation has satisfied most of the demands (Fig. 1a), production cost and yield remain suboptimal for most amino acids. Several amino acids, such as l-alanine and l-glycine, can only be enzymatically or chemically produced. High-performance fermentation strains are needed for overproducing the targeted amino acids^3^.

The traditional strategy for screening amino acid overproducers took the advantage of toxic analogues, which has similar size, structure, and charge properties as the proteinogenic amino acids. An analogue would compete with its corresponding amino acid for the finite tRNAs in the process of protein biosynthesis^4,5^. Once inserted into any polypeptide, the analogue could disrupt the synthesis or function of that polypeptide. The losing of functional proteins could result in growth retardation or even death^6^. Cells overproducing an amino acid might produce enough functional proteins to survive the stresses from the analogue of that amino acid^7^ and could be selected. For instance, a high concentration of 4-azaleucine has been successfully applied to select l-leucine overproduction strains^8–10^.

However, the use of analogues faces severe disadvantages that could compromise the selection results. First, an analogue could interfere with cellular activities beyond the protein synthesis. For example, it could jeopardize cell growth by disrupting the nucleus regions^7^, affecting the structure of cellular membranes^11^, inhibiting purine and pyrimidine biosynthesis or decreasing the level of ATP^7,11,12^. Thus, mutants that have enhanced amino acid productivities may not survive these side effects. Second, cells may escape from the selection pressure of an analogue by developing detoxification mechanisms. Specifically, the analogues could be blocked by amino acid transporters with increased selectivity, or pumped outside of the cells by enhanced efflux^6^ (Fig. 1b). The analogues could also be degraded to nontoxic forms or be incorporated into the bacterial proteome after evolution^13^. Therefore, it is necessary to test the selected mutations individually to verify their amino acid productivities. Third, proper analogues for specific amino acids are limited^5^. Therefore, it is of great need to develop alternative approaches that provide accuracy, sensitivity, and high-throughput simultaneously.

The 20 proteinogenic amino acids are encoded by 61 codons, and with up to six different codons specifying the same amino acid according to standard code table^14^. Many prokaryotes and eukaryotes display strong preference for certain codons over their synonymous alternatives^15,16^ (Fig. 1c). A codon is categorized as common or rare codon for a specific species, depending on the frequency of its occurrence in the coding DNA of the whole genome. This codon usage bias affects translation phases such as translation initiation, elongation, and protein folding^17–20^. Furthermore, the common and rare codons vary among different species, since a common codon for one species could be a rare one for another species^21^. A codon could be translated into a corresponding peptide-forming amino acid by a charged tRNA isoacceptor which pairs the codon with the amino acid.

The heterologous protein expressions in host strains are always challenging. The codons of heterologous proteins are generally not the favored ones in the host, and some are paired with cognate tRNAs with low abundancy^22–24^. Therefore, automated codon optimization algorithms have been developed to design coding sequences optimized for increasing expressions in certain hosts, which could lead up to 1000-fold increase in protein expressions^25^. Opposite to the codon optimization strategy, the heterologous protein expressions in hosts could theoretically be reduced by replacing common codons with synonymous rare alternatives, especially under amino acid starvation conditions. The charging levels of rare isoacceptors approached zero immediately after the cells were challenged by the amino acid starvation, while the ones of common isoacceptors remained high for a few minutes^24^. A rare isoacceptor is charged when its intracellular corresponding amino acid remains sufficient after charging the synonymous common isoacceptors^26^. Therefore, an unexplored approach for screening amino acid overproducers, which is supposed to have high intracellular amino acid concentrations, could be developed based on the natural competitions between rare and common isoacceptors for intracellular free amino acids.

For a gene with numbers of rare codons, its translation under amino acid starvation conditions could be maintained if the amounts of intracellular amino acids are sufficient to support the easy charging of the rare tRNAs (Fig. 1d). Such strains are likely to be the amino acid high production strains. By linking the protein expressions with cell growth or color formation, we could easily distinguish the high production strains from a pool of candidates. This approach overcomes the drawbacks of analogues by only targeting the reporter genes without affecting other cellular systems and biosynthetic processes.

In this study, we establish and apply the above strategy to obtain *Escherichia coli* and *Corynebacterium glutamicum* amino acid overproducers. Three amino acids, l-leucine, l-arginine, and l-serine, are chosen because they are important fermentation products and have specific rare codons. The system is constructed by replacing defined numbers of leucine, arginine, and serine codons with the corresponding *E. coli* rare codon CTA, AGG or TCC in antibiotic resistance protein (Kan^R^ or Spec^R^), green fluorescent protein (GFP) or the chromogenic prancerpurple protein (PPG, from ATUM). The proteins encoded by rare codon-rich derivatives of the genes are expressed at low levels under amino acid starvation or growth restriction conditions. We show that the protein expressions from the rare codon-rich gene derivatives are dramatically increased by feeding or enhanced intracellular synthesis of the corresponding amino acid. Therefore, amino acid overproducers are readily screened out and the conditions for screening or selection are optimized. To evaluate its performance, the above strategy is successfully applied to screen for l-leucine, l-arginine, and l-serine overproducers from *E. coli* mutation libraries. Several *C. glutamicum* strains overproducing l-arginine are also successfully selected by the same strategy. This study proves that our rare codon-based strategy is a promising alternative for the high-throughput screening of amino acid overproducers.

## Results

### Rare codon-based selection and screening systems

Here, we establish two systems for the identification of amino acid overproducers. One is a selection system based on rare codon-rich antibiotic resistance genes. Any strain that survives the antibiotics is likely an amino acid overproducer. The other is a screening system that used colored proteins encoded by genes harboring the rare codons. Overproducers of the targeted amino acids are readily identified by visual screening. The original leucine, arginine or serine codons of the marker genes were replaced by the rare synonymous CTA (0.39%), AGG (0.11%) or TCC (0.86%), individually^27^. Codon replacement was performed using PCR-based accurate synthesis and the generated genes were denoted RC (rare codon). The selection system was employed to pick out potential amino acid overproducers from *E. coli* mutation libraries derived by atmospheric room temperature plasma (ARTP) mutagenesis. For each candidate, titer of the targeted amino acid was verified by high-performance liquid chromatography (HPLC) and the desired strains were identified. To test the potentials of this system, this strategy was also employed to *C. glutamicum* by replacing the original l-arginine common codons of a selection marker gene by AGG (0.32%), the rarest arginine codon in *C. glutamicum*^27^.

### Effects of rare codon frequency on the selection system

**Fig. 2:**
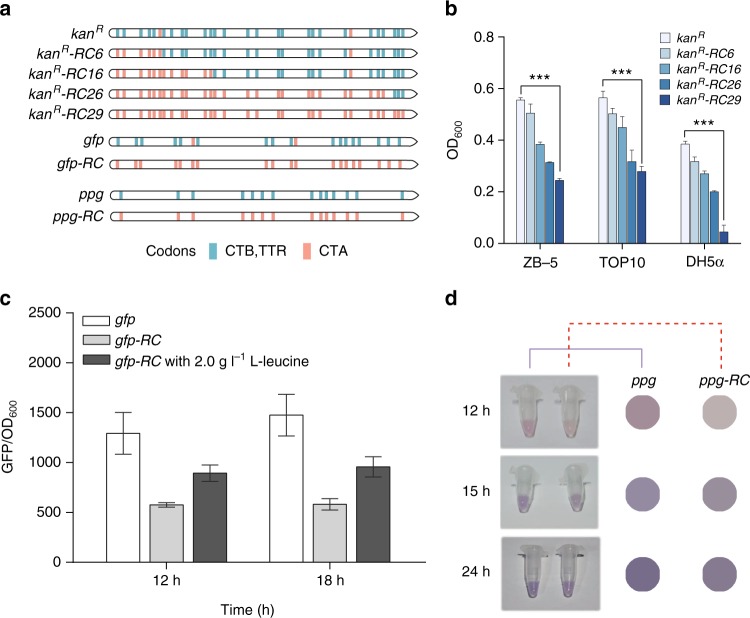
Effects of the frequency of leucine rare codon CTA on protein expressions. **a** Different numbers of the leucine codons on the wild-type *kan*^*R*^ were replaced by the rare one CTA, generating *kan*^*R*^*-RC6*, *kan*^*R*^*-RC16*, *kan*^*R*^*-RC26*, and *kan*^*R*^*-RC29*; the leucine codons on the wild-type *gfp* and *ppg* were also replaced by the rare alternative, generating *gfp-RC* and *ppg-RC*, respectively. **b** Influences of rare codon frequency on cell OD_600_ for *E. coli* strains harboring the rare codon-rich *kan*^*R*^ (***P < 0.001 as determined by two-tailed *t* test). **c** Effects of the incorporation of leucine rare codon CTA and l-leucine feeding on GFP expression. **d** Effects of the incorporation of leucine rare codon CTA on PPG expression as indicated by the differences in color development. Values and error bars represent the mean and the s.d. (*n* = 3)

The *kan*^*R*^ gene encoding the aminoglycoside 3′-phosphotransferase type Ia contains 29 leucine codons and was employed for the construction of the l-leucine selection system (Fig. 2a). To investigate the effect of rare codon frequency on protein expressions, a series of *kan*^*R*^ genes with a gradient frequency of rare codon replacement were examined for kanamycin resistance. The codon replacement, if needed, was done by synthesizing *kan*^*R*^ containing 6, 16, 26, and 29 leucine rare codon CTA, generating *kan*^*R*^*-RC6*, *kan*^*R*^*-RC16*, *kan*^*R*^*-RC26*, and *kan*^*R*^*-RC29*, respectively. The retarded Kan^R^ expression induced by rare codon would confer only limited resistance towards kanamycin, leading to arrested cell growth. These genes were introduced into *E. coli* strains DH5α and TOP10, as well as an l-valine overproduction strain ZB-5.

The M9 media with different carbon and nitrogen sources and the Luria−Bertani (LB) media with different dilution factors were tested in this study. In the presence of kanamycin, the 0.2× LB ensured significant differences in cell densities between strains harboring the wild-type *kan*^*R*^ and the *kan*^*R*^*-RC*s (Fig. 2b and Supplementary Fig. 1), while the M9 media did not lead to differentiations in strain growth. Compared with the wild-type *kan*^*R*^, the cell densities kept decreasing as the leucine rare codon on *kan*^*R*^ became more frequent, in agreeing with the assumption that translation efficiency correlated negatively with rare codon frequency, especially under nutrient limitation conditions. The largest difference in OD_600_ between DH5α strains containing wild-type *kan*^*R*^ and the *kan*^*R*^*-RC29* was up to 8.5-fold and was observed in the 18-h culture. Under the same experimental conditions, the largest differences in OD_600_ were 2.27-fold for ZB-5 and 2.02-fold for TOP10, respectively (Fig. 2b). The results indicate that the rare codon-inhibited protein expression is in a frequency-dependent manner.

### Feeding amino acids restored cell growth

**Fig. 3:**
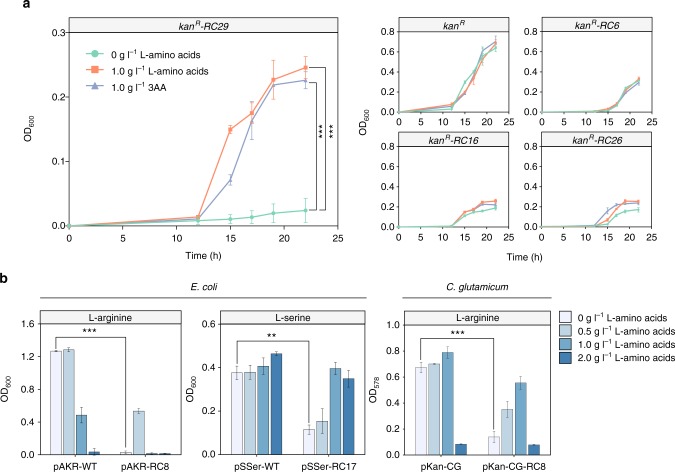
Cell growth restored by feeding the corresponding l-amino acids. **a** Effects of feeding l-leucine or a mixture of three amino acids (3AA: l-leucine, l-valine and l-isoleucine) on the cell growth for *E. coli* strains harboring *kan*^*R*^ genes with 6−29 leucine rare codons (*kan*^*R*^*-RC6*, *kan*^*R*^*-RC16*, *kan*^*R*^*-RC26*, *kan*^*R*^*-RC29*). **b** Changes in cell ODs after feeding l-arginine to *E. coli* and *C. glutamicum* strains harboring *kan*^*R*^ genes in which eight arginine codons were replaced by its rare alternatives (encoded by pAKR-RC8 or pKan-CG-RC8), and the growth restoration after feeding l-serine to *E. coli* strains carrying *spec*^*R*^ which was rich in serine rare codon (encoded by pSSer-RC17). Values and error bars represent the mean and the s.d. (*n* = 3). **P < 0.01, ***P < 0.001 as determined by two-tailed *t* test

The feeding assays were performed to investigate whether the increased intracellular concentrations of amino acids could alleviate the growth inhibition induced by the rare codons. The cell densities of DH5α strains containing *kan*^*R*^*-RC*s were partially restored by feeding l-leucine (1.0 g l^–1^) or a mixture of three amino acids (3AA, 0.3 g l^–1^ l-leucine, 0.3 g l^–1^ l-isoleucine, and 0.3 g l^–1^ l-valine) to the 0.2× LB medium (Fig. 3a). For strains containing *kan*^*R*^*-RC16*, the addition of l-leucine restored cell OD_600_ by 36.44%, and the addition of 3AA restored cell OD_600_ by up to 16.72%. The restoration percentages of feeding l-leucine and 3AA were 49.41 and 40.58%, respectively, for the strains carrying *kan*^*R*^*-RC26*. The strongest responses toward feeding were observed in strains containing *kan*^*R*^*-RC29*. At 22 h, the addition of l-leucine led to a tenfold OD_600_ increase from 0.024 to 0.246 and the addition of 3AA had a similar effect, leading to a ninefold OD_600_ increase. These results indicate that the growth restoration become stronger as the leucine rare codon on *kan*^*R*^ become more frequent.

To test the potential of the rare codon-based strategy, the *kan*^*R*^ and *spec*^*R*^ genes were also employed for the construction of the rare codon-based selection systems for selecting l-arginine and l-serine overproducers, respectively (Supplementary Note 1). Changing the last eight arginine codons to AGG inhibited cell growth in both *E. coli* and *C. glutamicum* (Supplementary Note 1). Compared to the *E. coli* carrying pAKR-WT, the OD_600_ of *E. coli* carrying pAKR-RC8 decreased 42.29-fold at 16 h (Fig. 3b). For *C. glutamicum* carrying pKan-CG-RC8, the OD_578_ decreased 5.35-fold at 16 h compared to the one carrying pKan-CG (Fig. 3b). The growth restoration was also investigated with a gradient of l-arginine addition. At 16 h, the OD_600_ of the *E. coli* strain carrying pAKR-RC8 was restored to 39.02% of the OD_600_ of the strain carrying pAKR-WT by adding only 0.5 g l^–1^ l-arginine (Supplementary Fig. 2a). Adding 1.0 g l^–1^ or higher concentrations of l-arginine inhibited the growth of *E. coli* strains. The cell density of *C. glutamicum* strains carrying pKan-CG-RC8 was restored to 49.72 and 60.53% of the cell density of the strain carrying pKan-CG by adding 0.5 g l^–1^ and 1.0 g l^–1^ l-arginine, respectively (Supplementary Fig. 2b). The results indicated the sensitivity of the Kan^R^ expression from pKan-CG-RC8 to the intracellular concentration of l-arginine. The repressed growth by rare codons and the growth restoration by amino acid feedings in both *E. coli* and *C. glutamicum* strains suggest the potential of applying this strategy to different organisms. In addition, the inhibited cell growth in *E. coli* was observed after replacing all serine codons in *spec*^*R*^ gene by rare codon TCC. The cell OD_600_ was restored to as much as 97.34% of the strains carrying pSSer-WT by adding only 1.0 g l^–1^ l-serine (Supplementary Fig. 2c). Taken together, these results indicate that extra amino acid feeding could restore the rare codon repressed protein expression partially or completely in our growth-based selection system.

### Modification of the selection stringency

**Fig. 4:**
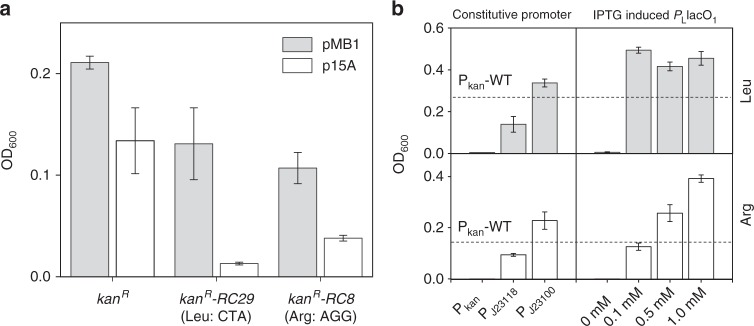
Selection stringency mediated by copy number and promoter strength. The *kan*^*R*^, *kan*^*R*^*-RC29*, and *kan*^*R*^*-RC8* represent the wild-type *kan*^*R*^, the *kan*^*R*^ containing leucine rare codon CTA, and the *kan*^*R*^ containing arginine rare codon AGG, respectively. **a** The effects of different ORIs on the cell OD_600_ for strains harboring the wild-type and the rare codon-rich markers. **b** The effects of constitutive and inducible promoters on the cell OD_600_ for strains harboring the rare codon-rich markers, the dashed line represents the cell OD_600_ for strain carrying the wild-type *kan*^*R*^ driven by its original promoter from pET-28a. Values and error bars represent the mean and the s.d. (*n* = 3)

To modify the selection stringency, the effects of gene copy number and promoter strength on our selection system were examined. The *kan*^*R*^ gene was driven by various well-characterized promoters and was placed on plasmids containing different origins of replication (ORIs). Strains containing the engineered plasmids were grown in 0.2× LB and the OD_600_ was measured. First, the original medium-copy-number ORI (pMB1) in pKan-RC29 or pAKR-RC8 was replaced by the low-copy-number ORI (p15A). The wild-type *E. coli* strains containing low-copy-number version of pKan-RC29 or pAKR-RC8 plasmid had 10.07- or 2.81-fold decrease in OD_600_ than the same strains containing the corresponding medium-copy-number version of pKan-RC29 or pAKR-RC8 plasmid under amino acid starvation conditions (Fig. 4a). Second, the original constitutive promoter *P*_kan_ was replaced by *P*_J23100_ (iGEM Part: BBa_J23100), *P*_J23118_ (iGEM Part: BBa_J23118), or *P*_L_lacO_1_ promoter^28^, respectively. *P*_J23100_ and *P*_J23118_ are constitutive promoters and the former one is more active^29^. *P*_L_lacO_1_ is the strongest promoter used in this study when fully induced by IPTG^29^. Since the cell growth correlated positively with the Kan^R^ level, significant differences in cell densities with different promoter replacements indicate that the expressions of Kan^R^ protein could be tuned by the strength of the promoters (Fig. 4b). When *P*_L_lacO_1_ was used, 0, 0.1, 0.5 or 1.0 mM of IPTG was added to the media to induce the expression of the promoter (Fig. 4b and Supplementary Fig. 3). The expressions of Kan^R^ increased with the increase of IPTG added to the media.

### Effects of rare codons on color screening system

The screening system used both the GFP and PPG. A total of 19 leucine codons in *gfp*, and 14 leucine codons in *ppg* were replaced by CTA, generating *gfp-RC* and *ppg-RC* (Fig. 2a). For the *gfp* system, difference in the fluorescence intensity between cells containing wild-type *gfp* and *gfp-RC* was measured (Fig. 2c). After 24 h, the fluorescence from cells containing the wild-type *gfp* was 52.16% higher than that from cells containing the *gfp-RC* and was 4.8 times as high as that of the control (empty vector) (Supplementary Fig. 4). Different amounts of l-leucine were added to the culture media to final concentrations of 0.5, 1.0 or 2.0 g l^–1^ to study the influence of amino acid feedings on GFP expressions. As expected, the number of fluorescent cells harboring *gfp-RC* increased steadily with the increase of l-leucine concentrations. Contrary to the wild-type *gfp*, the protein expression from *gfp-RC* relied on the feeding of l-leucine in a concentration-dependent manner (Supplementary Fig. 2d).

The negative effects of rare codons on the expression of PPG were reflected by the retarded color development. Since the expression of *ppg* was driven by *lac* promoter, a high concentration of IPTG (e.g. 1.0 mM) led to strong induction of the *ppg-RC* and counteracted the negative effects of rare codons. Therefore, weak induction was preferred to maximize the difference between protein expressions from the wild-type gene and its rare codon-rich derivatives. A concentration of 0.1 mM IPTG achieved clear discrimination in color development 12 h after the induction (Fig. 2d). Compared to the purple color developed from the wild-type *ppg*, the protein expression from *ppg-RC* gave the medium a light pink, indicating the inhibition effect of rare codons on the expression of PPG. The accumulation of colored protein in the 24-h culture of strains containing *ppg-RC* could still be distinguished from the wild-type *ppg* when 0.1 mM IPTG was added. The results demonstrate the stability and reliability of the rare codon-based screening system.

### Obtaining amino acid overproducers by the selection system

**Fig. 5:**
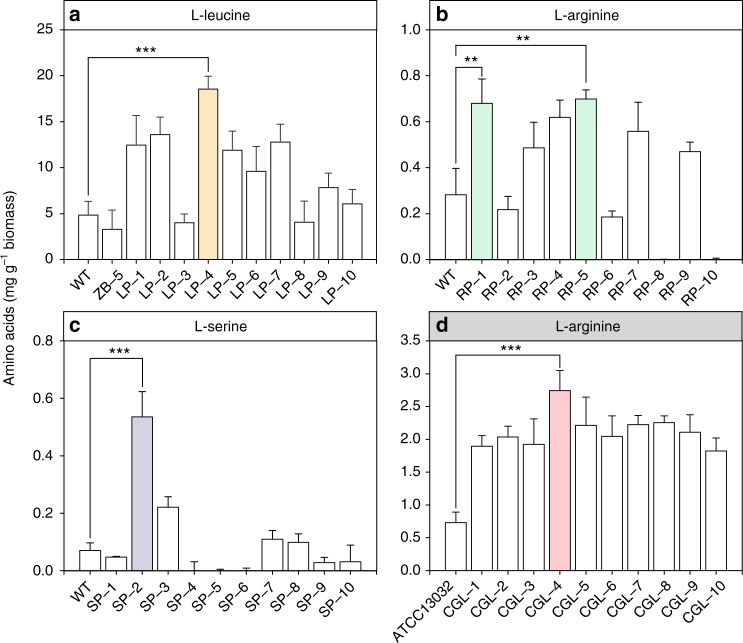
The amino acids produced by the wild-type and the mutated strains. **a** l-leucine productions of *E. coli* mutants selected by the leucine rare codon-rich *kan*^*R*^. **b** l-arginine productions of *E. coli* mutants selected by the arginine rare codon-rich *kan*^*R*^. **c** l-serine productions of *E. coli* mutants selected by the serine rare codon-rich *spec*^*R*^. **d** l-arginine productions of *C. glutamicum* mutants selected by the arginine rare codon-rich *kan*^*R*^. Values and error bars represent the mean and the s.d. (*n* = 3). **P < 0.01, ***P < 0.001 as determined by two-tailed *t* test

Besides uptaking amino acids directly from the environment, high intracellular concentrations of amino acids could be achieved through enhanced de novo productions. Therefore, this selection system could be used to select strains enabling enhanced de novo biosynthesis of l-leucine, l-arginine or l-serine. To verify the efficiency of the rare codon-based selection system, the plasmids pKan-RC29, pAKR-RC8 or pSSer-RC17 (Supplementary Data 1) were applied to random mutation libraries generated by ARTP. The ARTP mutated strains that survived the rare codon-based selection were isolated and cultured individually in 0.2× LB medium. After 12 h of incubation, the mutants selected by the leucine rare codon-based strategy achieved an average OD_600_ of 0.148 while the wild-type strain only grew to an OD_600_ of 0.032 (Supplementary Fig. 5). Eight out of the top ten mutants with the highest OD_600_ values (0.243–0.285) showed increased productivities for l-leucine (Fig. 5a). Among these strains, a strain (LP-4) with a titer of 18.55 mg l-leucine per gram biomass was achieved, which was 2.91 times higher than that of the wild-type strain. l-arginine overproducers RP-1, RP-5, and l-serine overproducer SP-1 were also selected by similar strategies (Supplementary Fig. 5). The titers of 0.679, 0.698, and 0.535 mg corresponding amino acids per gram biomass were achieved respectively (Fig. 5b, c). The system was successfully employed in obtaining *C. glutamicum* l-arginine overproducers. Among them, a mutated strain CGL-4 achieved 3.7-fold increase in l-arginine production (2.742 mg g^–1^ biomass) compared to that of the parent strain ATCC13032 (Fig. 5d). The selected overproducers could produce comparable amount of amino acid with and without the plasmid carrying the selection marker (Supplementary Fig. 6). Therefore, the presence of rare codon-rich marker genes has little effect on the production of the corresponding amino acid.

### Mechanisms of increased amino acid productions

**Fig. 6:**
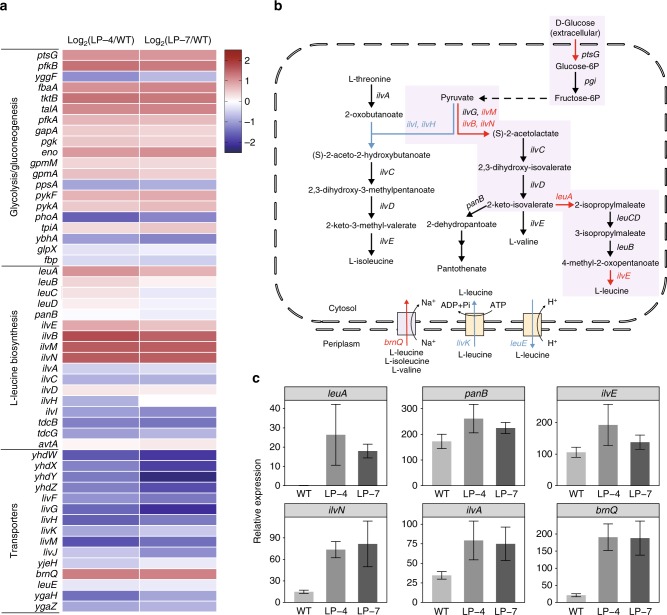
Transcription profiles of BCAA biosynthetic pathways in LP-4 and LP-7. **a** Transcriptome analysis of LP-4, LP-7, and the wild-type strains. Positive values (red) represent upregulated genes and negative values (blue) represent downregulated genes, which were calculated by the RPKM of LP-4 and LP-7 divided by that of the wild-type strain (in log_2_). The top, middle, and bottom panels contain genes in glycolysis, l-leucine biosynthesis and BCAA transportations, respectively. **b** The BCAA biosynthetic pathways in *E. coli*, red and blue arrows indicate the up- and downregulated genes, respectively. **c** qRT-PCR verification of the genes related to l-leucine biosynthesis and transportation. Values and error bars represent the mean and the s.d. (*n* = 3)

Significant differences in cell growth and amino acid productions were observed between the wild-type *E. coli* and the selected l-leucine overproducers. To figure out whether the increase in l-leucine production was due to the enhancement of biosynthetic pathways, the RNA sampled from cells in the exponential growth phase were sequenced for the wild-type strain and the selected mutants cultured in 0.2× LB. A total of over 27, 30, and 24 millions of raw reads were obtained for LP-4, LP-7, and the wild-type strains, respectively. The expression ratios of each gene involved in branched-chain amino acids (BCAAs) biosynthesis and transportations between the wild-type and LP-4 or LP-7 were calculated (Fig. 6a). A positive value denotes upregulated gene expression, while a negative value indicates downregulated gene expression.

l-leucine and l-valine biosynthesis share the same pathway from pyruvate to 2-keto-isovalerate (KIV) (Fig. 6b). LeuA catalyzes the first reaction in the l-leucine-specific biosynthesis pathway branching off from the l-valine pathway in *E. coli*. The coding gene *leuA* exhibited twofold higher expressions in both LP-4 and LP-7 compared to the wild-type strain, for which the activation was also verified by qRT-PCR analysis (Fig. 6c). The higher expression of *leuA* may enhance the conversion of KIV into 2-isopropylmaleate and hence l-leucine. The expression of *panB*, which is responsible for the competing pathway that channels KIV to pantothenate, remained the same level in all three strains. In addition, the biosynthesis of pyruvate, the key precursor of BCAAs, increased in both LP-4 and LP-7. Reduced amounts of pyruvate into other amino acid biosynthetic pathways could facilitate the distribution of pyruvate to the BCAA biosynthetic pathway. As shown by the transcription profiles, the expressions of *ilvM* and *ilvN* that encode the two small subunits of the first common enzyme (acetohydroxyacid synthase II) in the super pathways of BCAA biosynthesis were upregulated by 3.26- and 2.95-fold in LP-4, respectively, and 2.86- and 2.92-fold in LP-7, respectively. An average increase of 5.3-fold in *ilvN* expression and a slight increase of 1.5-fold in *ilvE* expression were also detected for the two strains by qRT-PCR, suggesting an enhanced flux of pyruvate towards l-valine and l-leucine biosynthesis. Taken together, the activation of l-leucine biosynthetic pathway enables the accumulation of intracellular l-leucine in the mutants.

The gene expressions related to l-leucine transportation decreased at varied degrees (Fig. 6a, b). Most notably, the gene expression levels of the general l-amino acid transporters *yhdW*, *yhdX*, *yhdY*, and *yhdZ* were downregulated in both strains by averages of 3.2-, 2.4-, 2.5-, and 2.4 fold, respectively. Gene expression levels of BCAA transporters *livH*, *livM*, *livG*, and *livF* were also downregulated in both strains by averages of 3.0-, 3.3-, 2.9-, and 2.1-fold, respectively. However, the expression of *brnQ*, a protein involved in amino acid uptake, was upregulated by 1.24- and 1.20-fold in LP-4 and LP-7, respectively, compared to the wild type. These results suggest that the intracellular accumulation of l-leucine in the selected overproducers could also be facilitated by increased l-leucine uptake and decreased l-leucine secretion, which is validated by the quantification of the mRNA levels of *brnQ* (Fig. 6c). Moreover, the expression of *rpoS* that encodes the stress response σ factor (σ^38^) was also increased twofold in both mutants, while its influence on the increased amino acid productions could not be determined.

To identify the specific mutations in l-leucine overproducers, the genomic DNA of the mutant strains LP-4 and LP-7 were sequenced by the illumine HiSeq PE150 platform, producing 8 million clean reads. A total of 56 and 62 scaffolds were generated for LP-4 and LP-7, respectively. The genomes were aligned among the mutants and the wild-type strain. The two mutants are highly similar and the largest difference between these two strains was a 7.5 kb duplicate of *arcA-yaaX* fragment that occurred only in LP-4. This indicates that the two l-leucine overproducers might derive from a common parent mutant but underwent slightly different DNA repairing processes^30^. Among the mutations related to amino acid accumulation (Supplementary Table 1), two are the pathway genes related to l-leucine biosynthesis (*ilvC* and *avtA*), one is the gene (*leuE*) that encodes l-leucine transporter and one is a leader peptide (*ivbL*). Mutations also occurred at the upstream regions for two upregulated genes (*brnQ* and *ptsG*).

For any of the LP-4, LP-7, and the wild-type strains, the amount of detected tRNA_3_^Leu^ in 1× LB medium was significantly higher than the one in 0.2× LB medium (Supplementary Fig. 7). About 96–99% of the tRNA_3_^Leu^ from the three strains were in the deacylated (uncharged) state in 0.2× LB medium, while tRNA_3_^Leu^ from the three strains were predominately in the aminoacylated (charged) state in 1× LB medium. In 0.2× LB medium, the amounts of detected tRNA_3_^Leu^ in LP-4 and LP-7, mostly in the deacylated state, were about 3.4- and 3.1-fold higher than the one of the wild-type strain, respectively. In 1× LB medium, the amounts of detected tRNA_3_^Leu^ in LP-4 and LP-7, mostly in the aminoacylated state, were about 30–40% higher than the one of the wild-type strain, respectively.

## Discussion

In this study, we establish a rare codon-based high-throughput screening or selection system. The introduction of one of the three types of rare codons (CTA, AGG or TCC) in target genes decreases the translation efficiency of the mRNA and the expressions of the targeted proteins, which could be partially restored through the addition or increased intracellular production of the corresponding amino acid (Fig. 3 and Supplementary Fig. 2). The protein expressions from rare codon-modified genes rely mostly on the intracellular concentration of the corresponding amino acid. The rare codon-based strategy only depends on a single modified gene sequence and has less influence on the expression of the proteome of the host. The efficiency of the strategy is rare codon frequency-dependent. When *kan*^*R*^ or *spec*^*R*^ harboring rare codons is applied to select for amino acid overproducers, several desired overproduction strains for l-leucine, l-arginine, and l-serine are successfully obtained from mutation libraries.

Here, the cell growth could be significantly affected by the promoter strength and the copy number of the *kan*^*R*^ genes in both rare codon-based selection systems for l-leucine and l-arginine. The different selective pressures could be achieved by adjusting the promoter activity, gene copy number (Fig. 4), or rare codon frequency (Fig. 2b). Since increased *kan*^*R*^ expression could significantly decrease the growth differences between cells harboring the wild-type marker gene and the rare codon-rich derivatives, weak promoters and relatively low copy number ORIs are conducive to the screening or selection process. The inducible promoter would adjust the screening pressure gradually at relatively low inducer concentrations, and an IPTG concentration of 0.1 mM was optimal in this study. However, adjusting the kanamycin concentration within the physiologically meaningful range did not result in notable difference in cell growth for strains harboring the wild-type and the rare codon-rich *kan*^*R*^. Nevertheless, the selectivity and sensitivity of the systems could be fine-tuned by varying the combination between promoters and ORIs.

Based on our assumption, all strains selected out on plates containing an antibiotic should be overproducers for the corresponding amino acid. However, false-positive strains were found in this study. For example, two out of the ten mutations selected by l-leucine selection system had lower l-leucine production levels than the wild-type strain had (Fig. 5a). On the other hand, six out of the ten mutations selected by l-serine selection system had lower l-serine production levels than the wild-type strain had (Fig. 5c). These mutants survived the pressure of antibiotic, but failed to overproduce the amino acid in the fermentation process. It turned out that the plasmids containing the selection markers were lost in those false-positive strains which obtained the antibiotic resistance through mutations in the host^31^.

The false-positive strains could be identified by integrating a *gfp* gene into the plasmids. The overproduction strains containing the plasmid could express fluorescent proteins and could be screened out. Alternatively, a dual-resistance plasmid, which contains a wild-type antibiotic resistance gene (e.g. ampicillin resistance gene) and a rare codon-modified resistance gene (e.g. *kan*^*R*^*-RC29*), could be used to eliminate the false-positive strains. Any strain surviving the dual antibiotic selection should contain the original plasmid and is likely an amino acid overproducer. Enhanced expressions of the aaRS or the rare tRNAs induced by mutagenesis could be another way for strains to escape the selection system. However, based on the genomic sequences of the two selected l-leucine overproducers, the coding and regulatory regions for both the aaRS and the rare tRNAs remained unchanged. The odds of mutations in these regions could be low for a selected amino acid overproducer.

Besides false-positive mutants, the toxicity of certain amino acid could also affect the results of selection. For instance, serine is among the most toxic amino acids and *E. coli* cells stop growing in the presence of only 106 mg l^–1^ l-serine^32^. The toxicity of serine could be alleviated by the addition of l-isoleucine^33^. The 0.2× LB used in this study might be able to partially counteract the serine toxicity, and the selected mutants showed l-serine productivities up to 7.3-fold of that of the wild-type strain (Fig. 5c). However, the productivities of the selected l-serine overproducers were still relatively low when compared with the l-leucine or the l-arginine overproducers. This could be attributed to serine toxicity, as strains producing high levels of l-serine could suffer from growth inhibition and die out. The amino acid productivities of the selected mutants also depend on other factors (Supplementary Note 2). Even so, the successful selection of mutants with increased productions for l-leucine, l-arginine, and l-serine demonstrates the sensitivity and efficiency of the rare codon-based selection strategy. Other interferences on the selection system might involve changes in the mRNA secondary structures of the marker genes due to rare codon replacement (Supplementary Fig. 8; Supplementary Note 3). Nevertheless, we prove that our system is applicable to different amino acids, as well as to other organisms such as *C. glutamicum*.

Besides the *gfp, kan*^*R*^, *spec*^*R*^ and *ppg* used in this study, numerous available selective markers or toxin−antitoxin systems, such as *sacB*^34^, *tolC*^35^ and *ccdB*^36^, could be used. If lethal genes would be used to select specific amino acid overproducer, the common codons in the corresponding antidote sequences should be replaced by rare codons. Thus, only strains with enhanced concentrations of intracellular amino acids could survive by maintaining the translation efficiency of the antidote genes to inhibit the lethal effects of the toxins. Furthermore, engineered tRNAs that recognize stop codons or carrying non-inherent amino acids might be needed to explore this strategy for amino acids without native rare codons^37,38^.

The transcriptome and qRT-PCR analysis show that the l-leucine overproducers have more active l-leucine biosynthetic pathway than the wild-type strain. The enhanced production of l-leucine enables the accumulation of intracellular l-leucine in the mutants, which could also be facilitated by increased l-leucine uptake and decreased l-leucine secretion. These strains could also survive the addition of l-leucine analogue (L-2-aminobutyric acid) of up to 1.5 g l^–1^ while the wild-type strain died at analogue concentrations >1.0 g l^–1^ (Supplementary Table 2). Among the mutations related to amino acid accumulation, the IvbL serves as a key component in the attenuation system that inhibits the expression of the *ilvBN* operon when the intracellular l-leucine and l-valine are abundant. Mutations in both its coding and upstream regions might alleviate its attenuation effect, leading to increased expressions of *ilvB* and *ilvN* as reflected by the transcription profile (Fig. 6). Although exploring the mechanisms related to amino acid overproduction is not our main goal, the genetic information should provide some preliminary clues for understanding our selected mutants. Besides, the activity of alanine aminotransferase (ALT, encoded by *alaA*) was also measured (Supplementary Fig. 9; Supplementary Note 4). However, the ALT activities in the selected overproducers do not exceed that of the wild-type strain, suggesting that the ALT alone could not significantly affect the l-leucine biosynthesis.

Taken together, we develop screening or selection systems for l-leucine, l-arginine or l-serine overproducers, respectively. Results show that the mRNA translations are inhibited by the integration of rare codons in the target genes in a frequency-dependent manner. As a proof-of-concept work, *E. coli* and *C. glutamicum* strains able to overproduce a specific amino acid are successfully selected from random mutation libraries. This work provides an alternative strategy to obtain amino acid overproducers.

## Methods

### Bacterial strains and culture conditions

The *E. coli* ZB-5 was an l-valine overproducer which was obtained by selection with norvaline, the l-valine analogue, from a mutation library of the *E. coli* strain JCL16, which was a derivative of *E. coli* wild-type strain BW25113^39^. The *E. coli* DH5α, TOP10 and ZB-5 used in this study were cultured in LB medium, and the *C. glutamicum* wild-type (ATCC13032) was cultured in CGIII medium. Strains employed in the selection system were cultured in 0.2× LB (2.0 g l^–1^ tryptone, 1.0 g l^–1^ yeast extract and 2.0 g l^–1^ NaCl) for *E. coli* and 0.3× CGIII (3.0 g l^–1^ peptone, 3.0 g l^–1^ yeast extract, 8.0 g l^–1^ NaCl and 0.6% glucose) for *C. glutamicum*. Solid media was prepared in all cases by adding 1.5% (w/v) agar. The *E. coli* strains were routinely grown in broth or on plates supplemented with appropriate antibiotics at the following concentrations: 50 μg ml^–1^ of kanamycin (25 μg ml^–1^ for *C. glutamicum*), 100 μg ml^–1^ of ampicillin, 25 μg ml^–1^ of chloramphenicol or 50 μg ml^–1^ of spectinomycin. The *E. coli* and *C. glutamicum* strains were grown at 37 and 30 °C, respectively.

### Rare codon introductions

The codon usages of the *E. coli* and *C. glutamicum* genomes were generated from protein coding sequences according to Refseq^40^. The rare codon CTA and TCC were chosen for the selection of l-leucine and l-serine overproducers in *E. coli*, respectively. The rare codon AGG could be used for selecting l-arginine overproducers in both *E. coli* and *C. glutamicum*. All these rare codon-modified genes were synthesized by PCR-based accurate synthesis^41^ (Supplementary Data 2).

### Plasmid constructions

All the DNA manipulations and cloning for system constructions were performed by standard cloning techniques^42^. The plasmids and primers used in this study are listed in Supplementary Data 1 and 3, respectively. To construct pKan-RC29, pCm-GFP-RC, and pCPB-37-441-RC (encoding *ppg*), leucine codons in these marker genes were all replaced by rare codon CTA. The genes were synthesized by ~60 bp oligonucleotides with 20 bp overlaps and cloned into the corresponding vectors (pKan-F/pKan-R for pKan-RC29, pCm-F/pCm-R for pCm-GFP-RC, and CPB-F/CPB-R for pCPB-37-441-RC) by Gibson assembly^43^. To construct pKan-RC6, pKan-RC16, and pKan-RC26, the fragments containing 6, 16, and 26 rare codon CTAs were amplified from pKan-RC29 using primers 6RC-F/6RC-R, 16RC-F/16RC-R, and 26RC-F/26RC-R, and cloned into pKan vector. The *E. coli-Corynebacterium* shuttle vector pKan-CG-RC8 was derived from pXMJ19, of which the *cat* marker was replaced by *kan*^*R*^. The different promoters were replaced by in vivo assembly^44^.

### Rare codon-based selection system

The pKan, pKan-RC6, pKan-RC16, pKan-RC26, and pKan-RC29 were transformed into *E. coli* DH5α, TOP10 and ZB-5. For each strain, three colonies were randomly picked and inoculated respectively into 15 ml 0.2× LB medium supplemented with kanamycin (50 μg ml^–1^) and incubated at 37 °C in a shaker at 200 rpm. The OD_600_ were measured at defined time points. The DH5α strains harboring pKan, pKan-RC6, pKan-RC16, pKan-RC26, and pKan-RC29 were inoculated respectively into 10 ml 0.2× LB medium with l-leucine (1 g l^–1^) or 3AA (l-leucine, l-isoleucine, and l-valine, 0.3 g l^–1^ for each amino acid) for each group. The l-arginine and l-serine selections using pAKR-RC8 and pSSer-RC17 were processed in the same way, while the l-arginine and l-serine were replenished at 0.5, 1.0, and 2.0 g l^–1^ respectively. The OD_600_ (OD_578_ for *C. glutamicum*) were measured in triplicate at defined time points.

### Rare codon-based screening system

The pCm-GFP and pCm-GFP-RC were transformed into *E. coli* DH5α. For each strain, three colonies were randomly picked and inoculated respectively into 10 ml LB medium and incubated at 37 °C in a shaker at 200 rpm, while the background fluorescence was measured using DH5α harboring pCm. The fluorescence (excitation: 470 nm; emission: 510 nm) and OD_600_ were measured at 12 h. The fluorescence microscopy images were captured at 12, 18, and 24 h from drops of culture solution containing fluorescent cells by a Nikon Eclipse 80i system equipped with DS-Ri1 camera. The ratio of GFP to OD_600_ (GFP/OD_600_) was used to represent fluorescence intensity. The DH5α strain harboring plasmids pCPB-37-441 or pCPB-37-441-RC was inoculated into 10 ml LB medium containing 0.1 mM IPTG and incubated in a shaker at 200 rpm at 37 °C.

### Screening amino acid overproducers from mutation libraries

The ARTP mutation system (Wuxi Tmaxtree Biotechnology Co., Ltd.) that could cause greater gene damage than traditional mutagenesis was employed to generate the mutation libraries^45^. The wild-type strain grown to OD_600_ between 0.5 and 0.8 was selected for ARTP treatment. Ten microliters of the culture were dipped onto the stainless-steel minidisc and then exposed to ARTP jet for 0, 20, 40, and 60 s with fatal rates of 0, 42, 65, and 79%, respectively. After the treatment, mutants were washed into sterilized 1.5 ml Eppendorf tube with 200 μl LB medium and incubated in a shaker at 200 rpm at 37 °C for 1 h. Then 100 μl of the culture was inoculated into 5 ml LB medium and incubated at 250 rpm at 37 °C until the OD_600_ reached 0.4–0.6. The *E. coli* ARTP mutants were made into competent cells and transformed with pKan-RC29, pAKR-RC8, pSSer-RC17 for l-leucine, l-arginine, and l-serine overproducers selection, respectively. While the *C. glutamicum* mutants were transformed with pKan-CG-RC8 via electroporation^46^. The fast growing cells in 0.2× LB in the presence of the corresponding antibiotic were enriched and selected. The selected mutants were then inoculated into 20 ml M9 medium (CGXII for *C. glutamicum*) with 4% glucose and the concentrations of l-leucine, l-arginine, and l-serine were measured at 24 h using a HPLC system (Shimadzu, Tokyo, Japan).

### Analysis of aminoacylation levels of tRNA

Total tRNA was isolated using TRIzol reagent (Invitrogen) and redissolved in 10 mM sodium acetate (pH 4.5). The aminoacylation levels of tRNA were evaluated for the l-leucine rare codon CTA. For each sample, a total of 4.0 μg RNA was mixed with 2× loading buffer containing 8 M urea, 0.25% (w/v) bromophenol blue and 0.25% (w/v) xylene cyanol dissolved in 300 mM sodium acetate (pH 5.2). The charged and uncharged tRNAs were separated by acid polyacrylamide gel prepared with 0.1 M sodium acetate (pH 5.2). The concentration of the gel was 6.5% (50 cm in length) or 17% (22 cm in length) and the electrophoresis was performed at 220 or 110 V, respectively, until the bromophenol blue reached the bottom of the gel.

The gel between xylene cyanol and bromophenol blue was blotted onto a positively charged nylon membrane under 23 V at 4 °C for 1.5 h. The tRNA was crosslinked to the membrane at 0.12 J of UV light and dried at 80 °C for 30 min. Prehybridization was carried out at 42 °C for 2 h in hybridization solution containing 6× SSC, 5× Denhardt’s, 0.5% (m/v) SDS and 100 μg ml^–1^ of sheared, denatured salmon sperm DNA. Hybridization was then performed in the same solution at 42 °C overnight with ^32^P-labled radioactive probe (Supplementary Data 3). The membrane was washed three times in 3× SSC, 0.25× Denhardt’s, 5% SDS and 25 mM Na_2_HPO_4_ at 42 °C. The radioactivity present in specific bands was measured using a phosphorimager scanner.

### cDNA library preparation and sequencing

The *E. coli* l-leucine overproducers LP-4 and LP-7 and the parent strain cultured in 0.2× LB were harvested at the exponential phase. Total RNA was isolated using TRIzol reagent (Invitrogen) and residual DNA was removed from the extracted RNA by RNase-free DNase (Thermo Fisher, RapidOut DNA Removal Kit). The mRNA was fragmented and cDNA was synthesized using the mRNA fragments as templates by the NEBNext Ultra Directional RNA Library Prep Kit for Illumina (New England Biolabs). Short fragments were purified and resolved with elution buffer for end reparation and single nucleotide A (adenine) addition. After agarose gel electrophoresis, the fragments between 150 and 200 bp were selected and ligated to sequencing adaptors using the USER Enzyme (New England Biolabs) at 37 °C for 15 min and amplified by PCR. The Agilent 2100 Bioanaylzer and ABI StepOnePlus Real-Time PCR System were used for the qualification and quantification of the sample library. The library was sequenced using Illumina HiSeq^TM^ 2000 platform.

The raw reads were filtered into clean reads and aligned to the reference sequences using SOAPaligner/SOAP2. The aligned data were utilized to calculate the distribution of reads on reference genes and to perform coverage analysis.

### Quantification of the mRNA level by qRT-PCR

The *E. coli* wild-type strain and the selected l-leucine overproducers were harvested at exponential phase. Total RNA was isolated using RNeasy Mini Kit (Qiagen) and the residual DNA was digested using Qiagen RNase-Free DNase Set. After reverse transcription, mRNAs of the selected genes were quantified by qRT-PCR using a Roche LightCycler 96 System with SYBR Green I detection. The housekeeping gene *cysG* was used as reference as it tends to maintain steady expression upon enzyme overexpression under various growth conditions^47^. Quantitative PCR was performed using SYBR Premix DimerEraser (TaKaRa) with an initial denaturing at 95 °C for 30 s, followed by 40 cycles of 5 s at 95 °C, 30 s at 55 °C and 25 s at 72 °C. Melting curve analysis was performed by raising the temperature from 60 to 95 °C at a rate of 0.1 °C s^–1^, with five signal acquisitions per degree. Data were acquired from three biological replicates and each sample was measured in duplicate.

### Genome sequencing

The genomic DNA was extracted from cells harvested at the exponential growth phase. The harvested DNA was detected by agarose gel electrophoresis and quantified by Qubit. The whole-genome sequencing was performed on the Illumina HiSeq PE150 platform. Raw reads containing more than 40 low-quality bases (Q score ≤ 38) were filtered out and the clean reads were assembled using SOAPdenovo (version 24)^48^. The coding genes, repetitive sequences, and noncoding RNA were predicted using GeneMarkS (version 4.17)^49^, RepeatMasker (version 4.0.5)^50^, tRNAscan-SE (version 1.3.1)^51^, and rRNAmmer (version 1.2)^52^. The coding regions were annotated by DIAMOND^53^ with E-value ≤ 1e^–5^ according to the NR, KEGG, COG and the Swiss-Port databases.

Comparative genomic analysis including the SNV and InDel annotations were analyzed using MUMmer (version 3.0)^54^ and LASTZ (version 1.02)^55^. Mutations occurring in the CDSs and intergenic regions of LP-4 and LP-7 related to amino acid overproductions were amplified and confirmed by Sanger sequencing (Supplementary Data 3).

## Electronic supplementary material

Supplementary Information

Descriptions of Additional Supplementary Files

Supplementary Data 1

Supplementary Data 2

Supplementary Data 3

## Data Availability

Data associated with this project can be found at the NCBI under BioProject PRJNA471786. The sequence data have been deposited in the SRA database under the study SRP148008. The authors declare that the data are available from the authors upon request.
