# Supplementary material for: Utilization of rare codon-rich markers for screening amino acid overproducers
Source: Nat Commun. 2018 Sep 6;9:3616. doi: 10.1038/s41467-018-05830-0 (PMC6127279; doi:10.1038/s41467-018-05830-0)
Supplement: Supplementary file 1 — Supplementary Information [file 41467_2018_5830_MOESM1_ESM.docx]

**Utilization of rare codon-rich markers** **for screening amino acid overproducers**

Zheng *et al*.

**Supplementary Note 1**

To generalize the strategy, the *spec^R^* that encodes the aminoglycoside adenylyltransferase was used for the establishment of L-serine selection marker (Supplementary Data 2). The rare codon AGG could be used for selecting L-arginine overproducers in both *E. coli* and *C. glutamicum*. Perhaps due to the Shine-Dalgarno-like feature of the rarest arginine codon AGG^1^, having 16 arginine codon AGG in the *kan^R^* gene could cause irreversible inhibition on Kan^R^ expression, as reflected by ceased cell growth in 1 × LB medium even when L-arginine was added. Thus, only a subset of the arginine codons on *kan^R^* was replaced. For the wild-type *kan^R^*, the second arginine codon happens to be the rare one AGG. To avoid confusion between the introduced and the native rare codons, the replacement was performed from the 3’-end of the *kan^R^* gene (Supplementary Data 2).

**Supplementary Note 2**

The increase in amino acid productions depends on the characteristics of the initial strain, the approach and round of mutagenesis, the size of the mutation library and the fermentation conditions. In this study, only one round of mutagenesis was employed on the wild-type *E. coli* strain. Thus, it would be impractical to expect a dramatic increase in yield that outcompetes the industrial strains, which have been engineered extensively for amino acid overproductions.

**Supplementary Note 3**

The replacement of leucine rare codon CTA in *kan^R^* maintains the protein sequence, but not the mRNA sequence. The secondary structure of mRNA could be predicted by its minimum free energy (MFE)^2^. The replacement of leucine common codons by CTA has little effects on the GC content of *kan^R^* nucleic acid sequence, and the change in MFE is less than 7%. This variation is not big enough to disrupt the function of mRNA for translation, but could lead to a significant change of the secondary structure of the mRNA (Supplementary Fig. 8). The secondary structures of the mRNAs of *kan^R^*-*RC6*, *kan^R^-RC16*, *kan^R^-RC26* and *kan^R^-RC29* were similar, but are different from the one of the wild-type *kan^R^*. This may contribute to the phenomenon that the growth retardation caused by 6–29 rare codon replacement could only be partially restored to a similar level below that of the wild-type control (Fig. 3a).

**Supplementary Note 4**

Alanine aminotransferase (encoded by *alaA*) catalyzes the reversible transfer of an amino group from L-alanine to 2-ketoglutaric acid, producing pyruvate and L-glutamate. The pyruvate is the precursor of L-leucine, and L-glutamate serves as the amino group donor for most amino acids including L-leucine. The alanine aminotransferase activity was determined by a coupled enzymatic reaction converting pyruvate to L-lactate at the expense of one molecule of NADH. The consumed NADH was detected by the decrease in absorbance at 340 nm. The decreasing rate of absorbance is proportional to the activity of alanine aminotransferase.


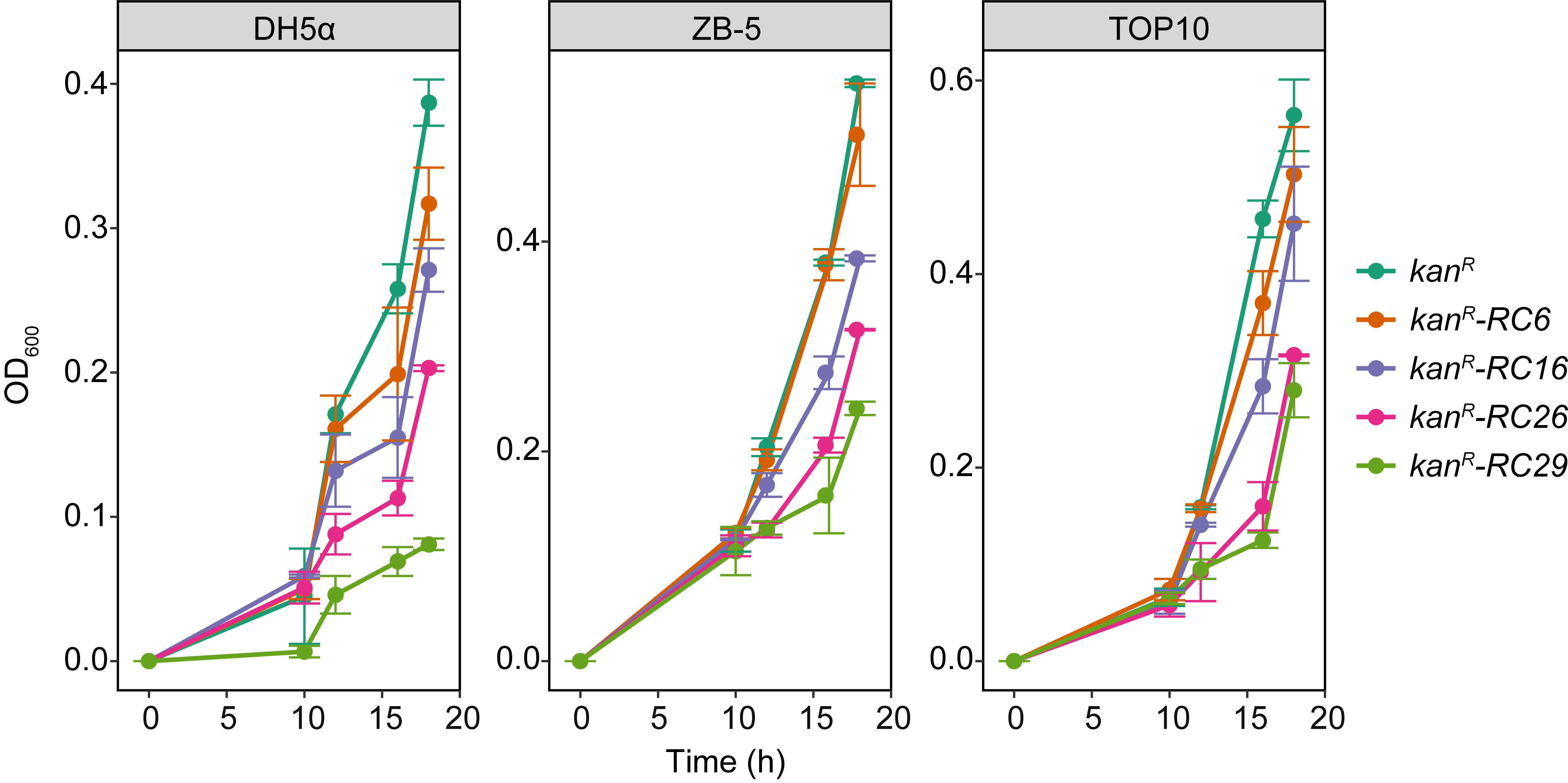


**Supplementary Figure 1. Growth curves of strains harboring rare codon-rich *kan^R^*.** The *E. coli* DH5α, ZB-5 and TOP10 strains harboring *kan^R^* genes with 6 to 29 of rare leucine codon CTA (*kan^R^-RC6*, *kan^R^-RC16*, *kan^R^-RC26*, *kan^R^-RC29*) were cultured in diluted culture medium (0.2 × LB). Values and error bars represent the mean and the s.d. (*n*=3).


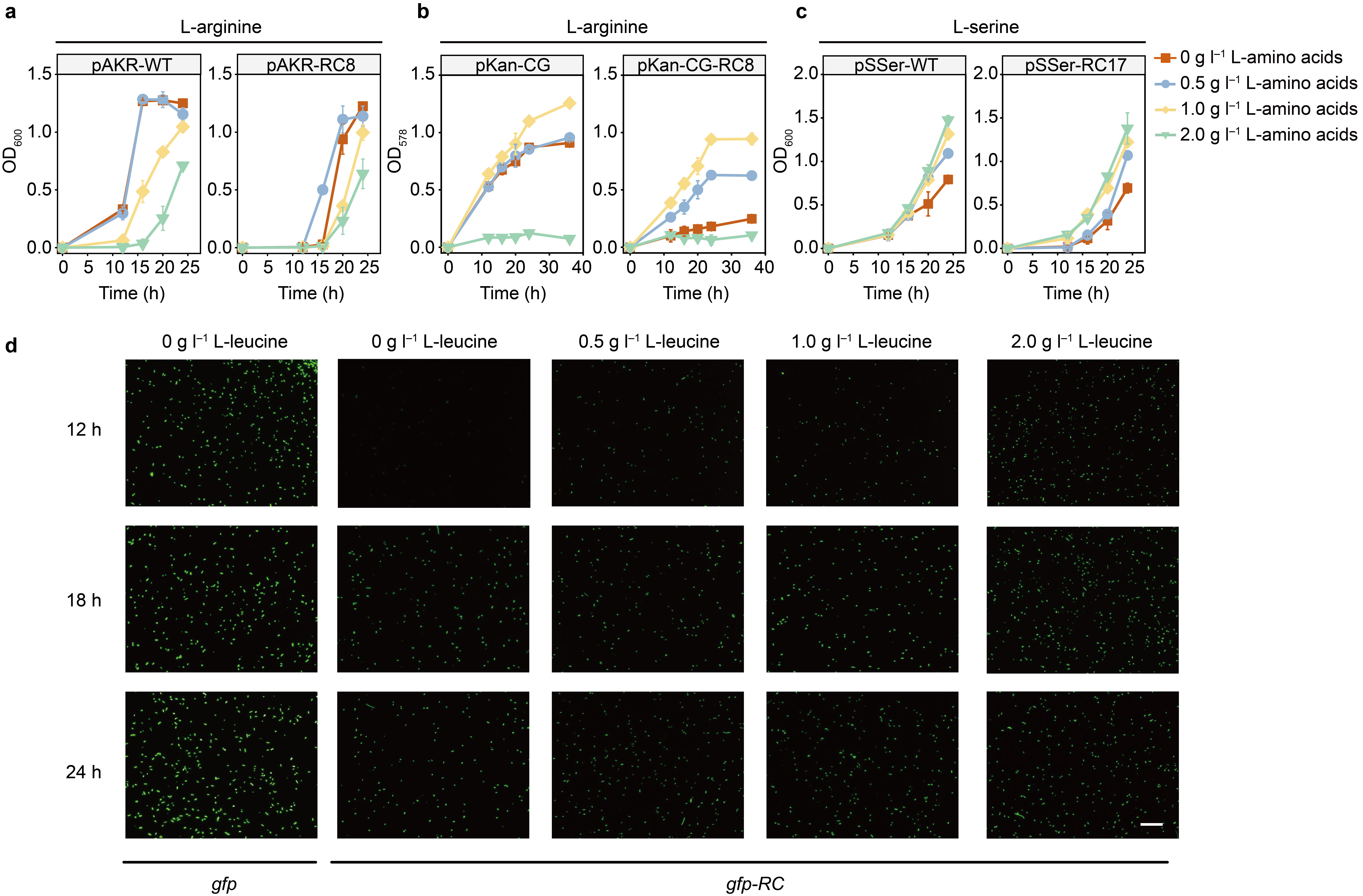


**Supplementary Figure 2.** **Growth restorations by feeding the corresponding L-amino acids**. Feeding L-arginine could restore the ODs of *E. coli* (**a**) or *C. glutamicum* (**b**) strains harboring *kan^R^* which was rich in arginine rare codon AGG (encoded by pAKR-RC8 or pKan-CG-RC8). Feeding L-serine (**c**) could restore the ODs of *E. coli* strains harboring *spec^R^* which was rich in serine rare codon TCC (encoded by pSSer-RC17). The fluorescence microscopy images (**d**) of cells harboring the wild-type *gfp* and the rare codon-rich *gfp* (*gfp-RC*) after the addition of L-leucine. Scale bar, 20 μm. Values and error bars represent the mean and s.d. (*n*=3).


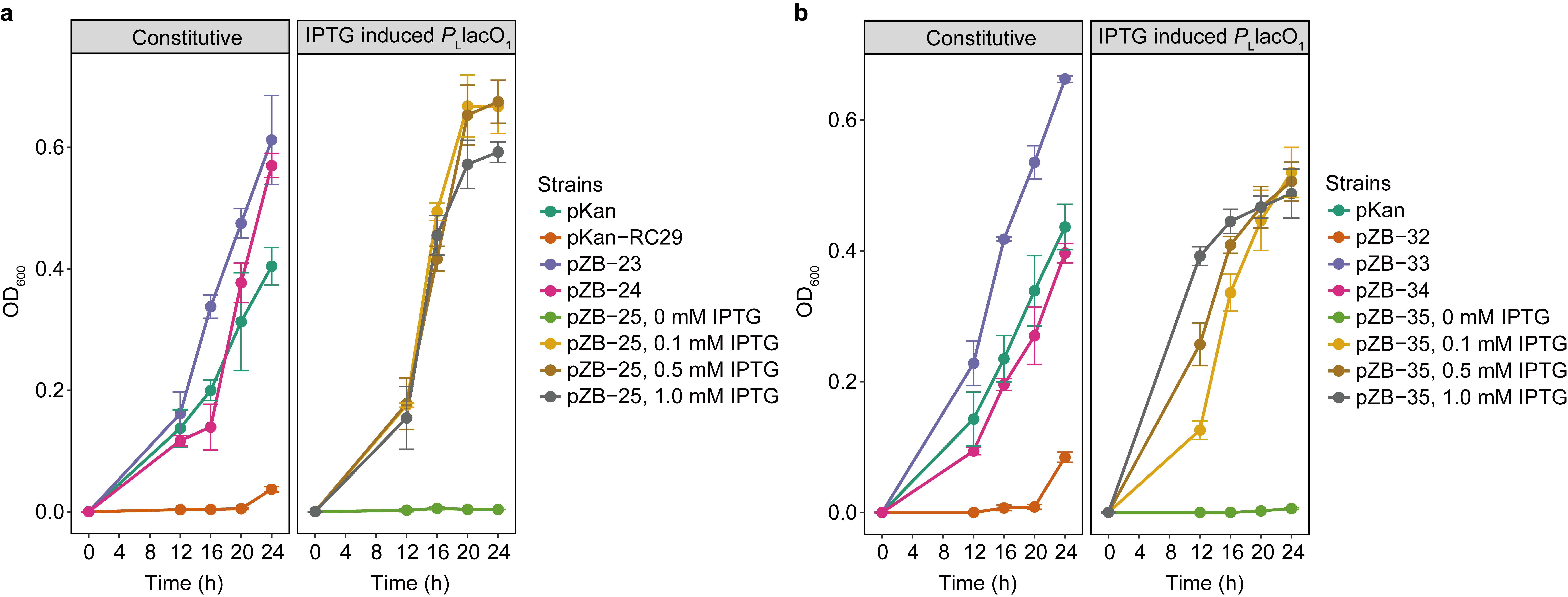


**Supplementary Figure 3. Effects of promoter strength on the selection stringency.** The OD_600_ of *E coli* strains harboring *kan^R^* genes rich in either leucine rare codon CTA (**a**) or arginine rare codon AGG (**b**) driven by either constitutive or inducible promoters. The constitutive promoters used here were the *kan^R^* promoter from vector pET-28a (in pKan, pKan-RC29 for L-leucine and pZB-32 for L-arginine), the strong promoter *P*_J23100_ (in pZB-23 and pZB-33) and the weak promoter *P*_J23118_ (in pZB-24 and pZB-34). The *P*_L_lacO_1_ promoter used here was induced by 0–1.0 mM IPTG (in pZB-25 and pZB-35). Values and error bars represent the mean and s.d. (*n*=3).


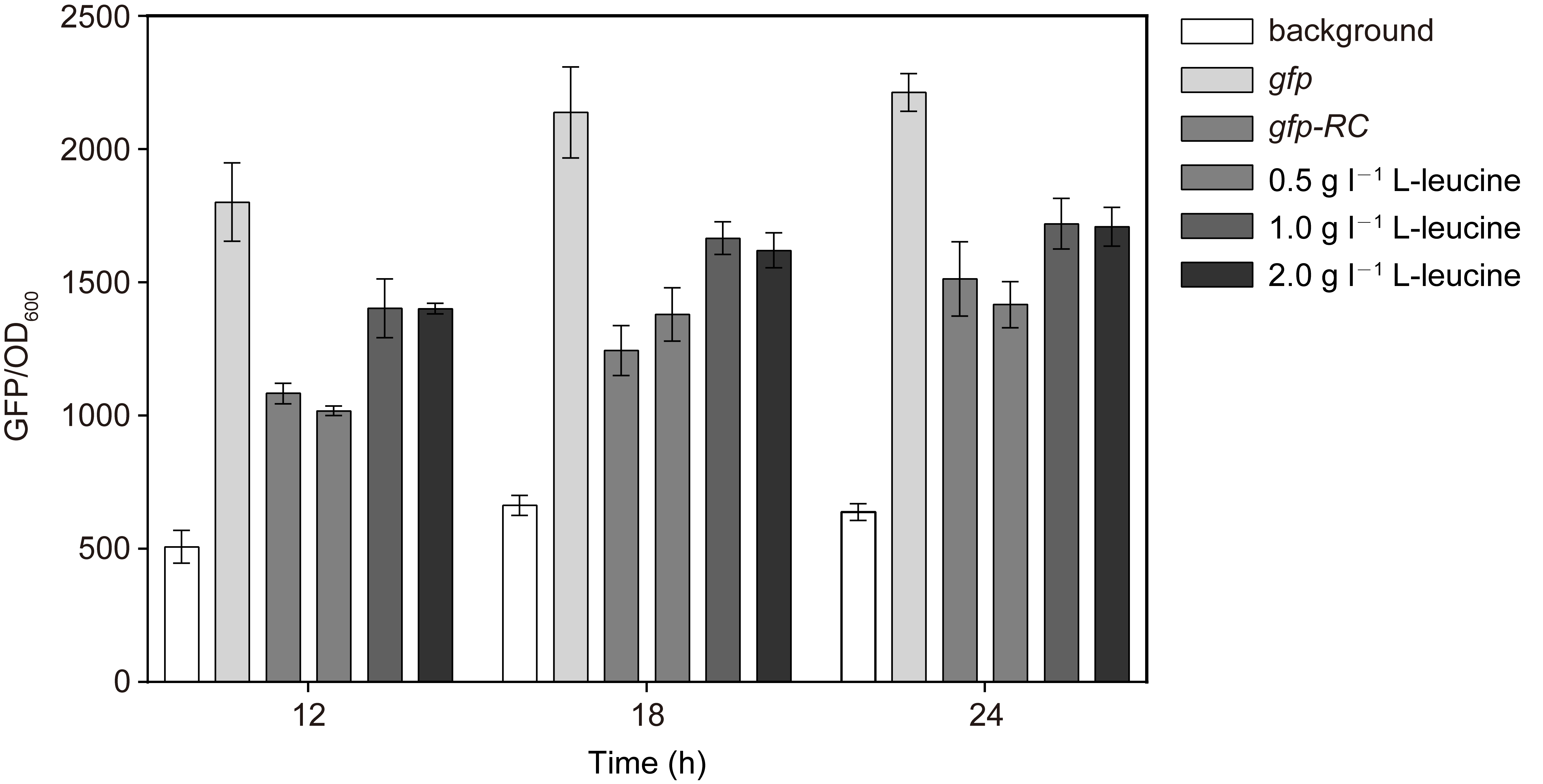


**Supplementary Figure 4.** **Changes in fluorescence intensity.** Replacing the leucine codons in *gfp* with the rare alternative CTA (*gfp-RC*) decreased the fluorescence intensity, which could be restored by extra feeding of L-leucine. Values and error bars represent the mean and the s.d. (*n*=3).


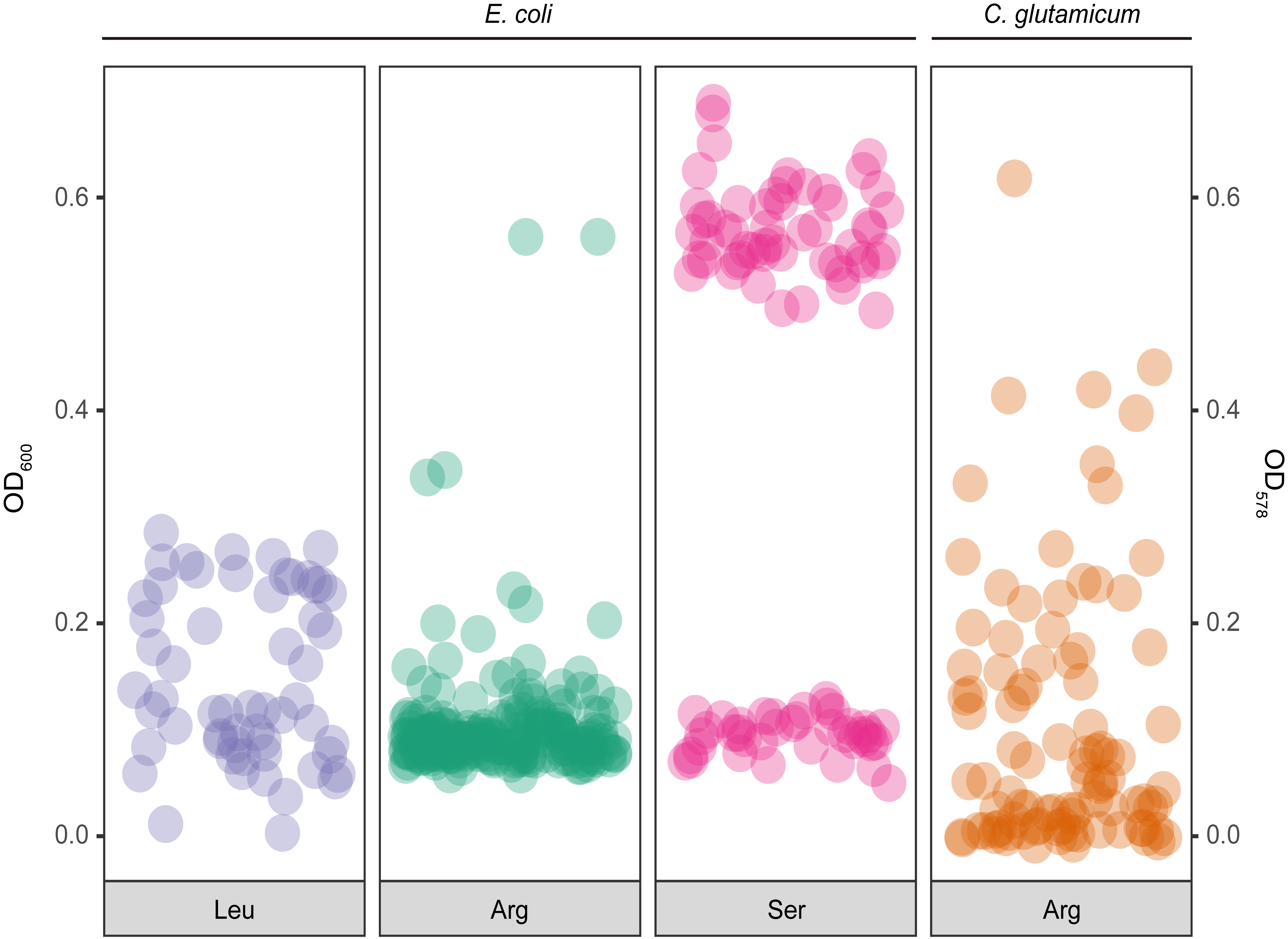


**Supplementary Figure 5****. Growth of mutants selected by rare codon-rich markers.** The *E. coli* mutants were selected by a *kan^R^* gene which was rich in leucine rare codon CTA or arginine rare codon AGG, or a *spec^R^* gene which was rich in serine rare codon TCC. The *C. glutamicum* mutants were also selected by a *kan^R^* gene which was rich in arginine rare codon AGG.


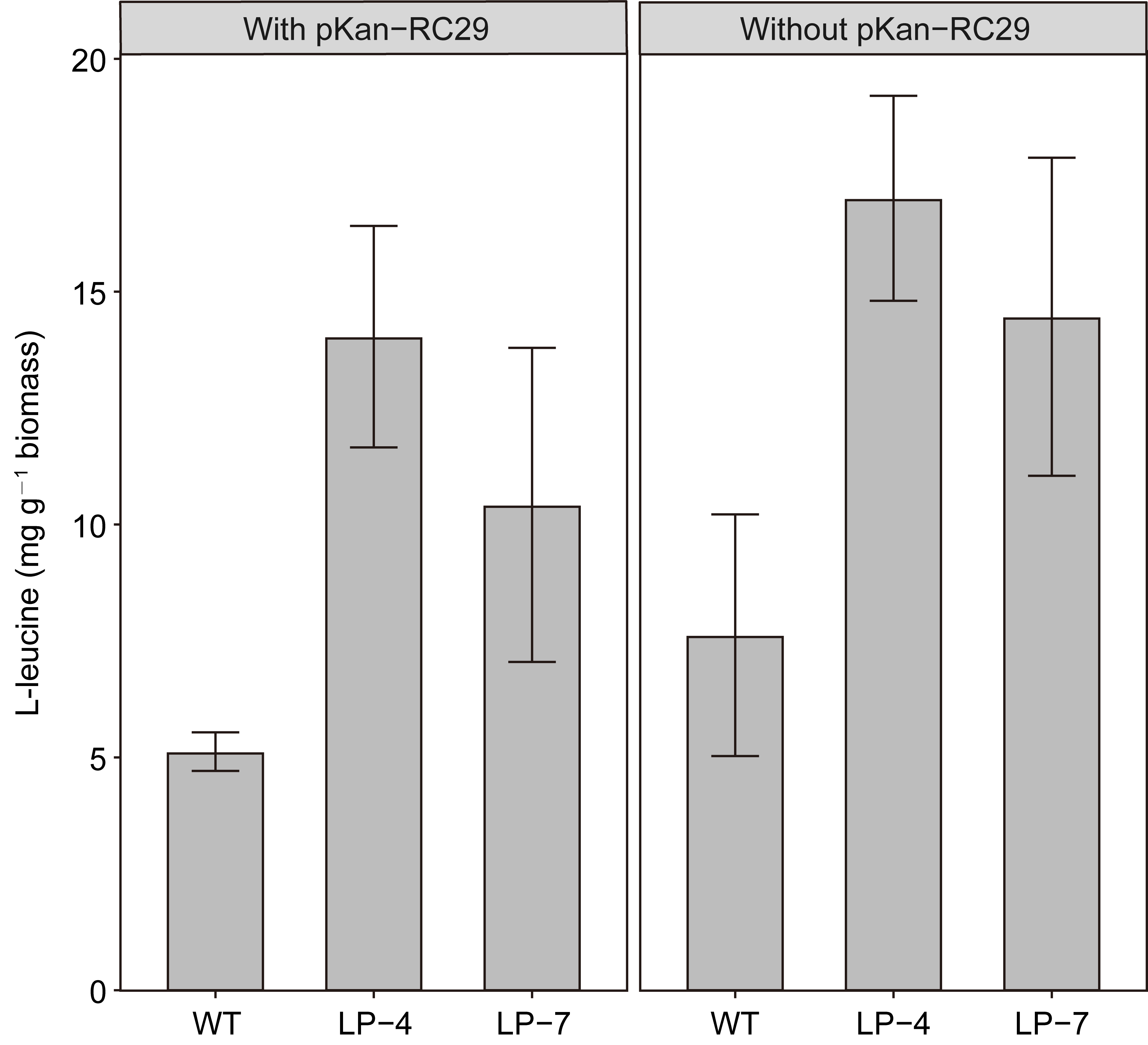


**Supplementary Figure 6. The L-leucine produced by the selected mutants.** The enhanced L-leucine productions by both LP-4 and LP-7 were verified with (left) and without (right) the rare codon-rich selection plasmids. Values and error bars represent the mean and s.d. (*n*=3).


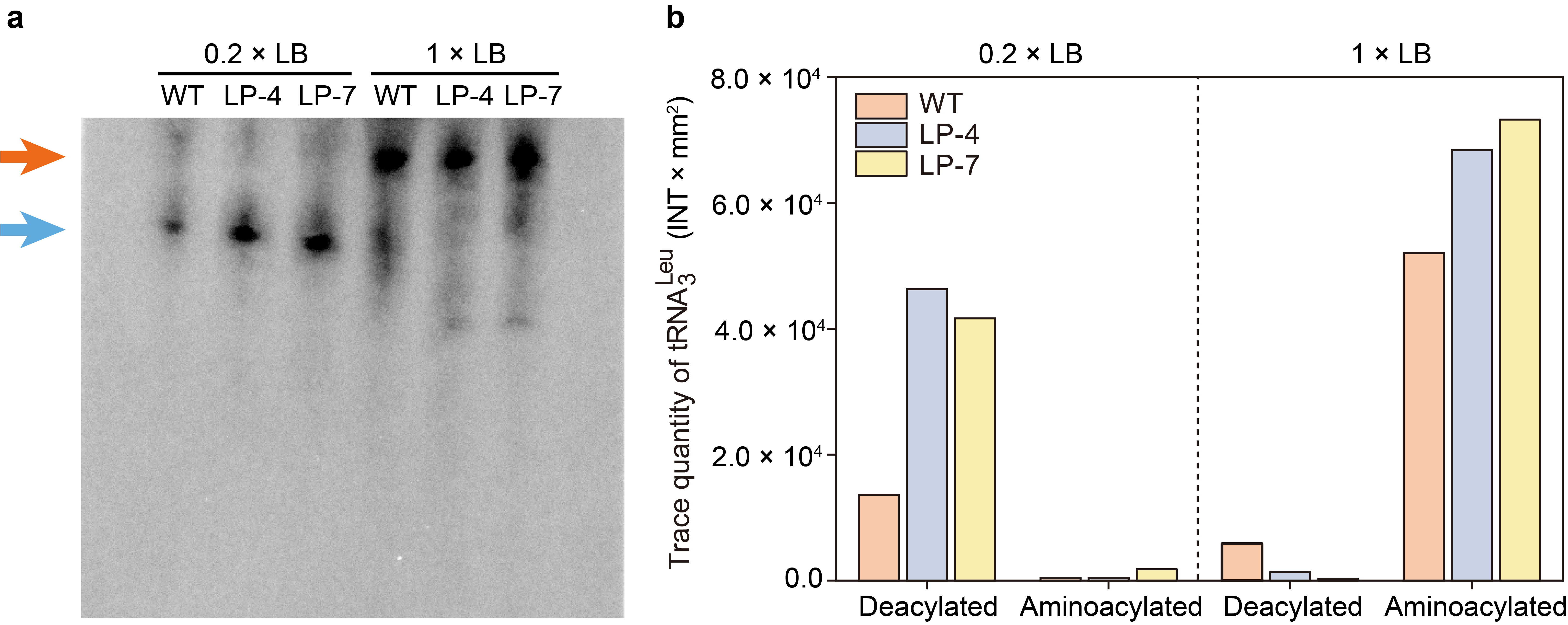


**Supplementary Figure 7. The amount and charging levels of** $\text{tRNA}_{\text{3}}^{\text{Leu}}$**.** The total tRNA of LP-4, LP-7 and the wild-type strains was extracted from cells grown in both 0.2 × LB and 1 × LB media. The charged (red arrow) and uncharged (blue arrow) forms of $\text{tRNA}_{\text{3}}^{\text{Leu}}$ were separated by acid gel (**a**) and quantified (**b**).


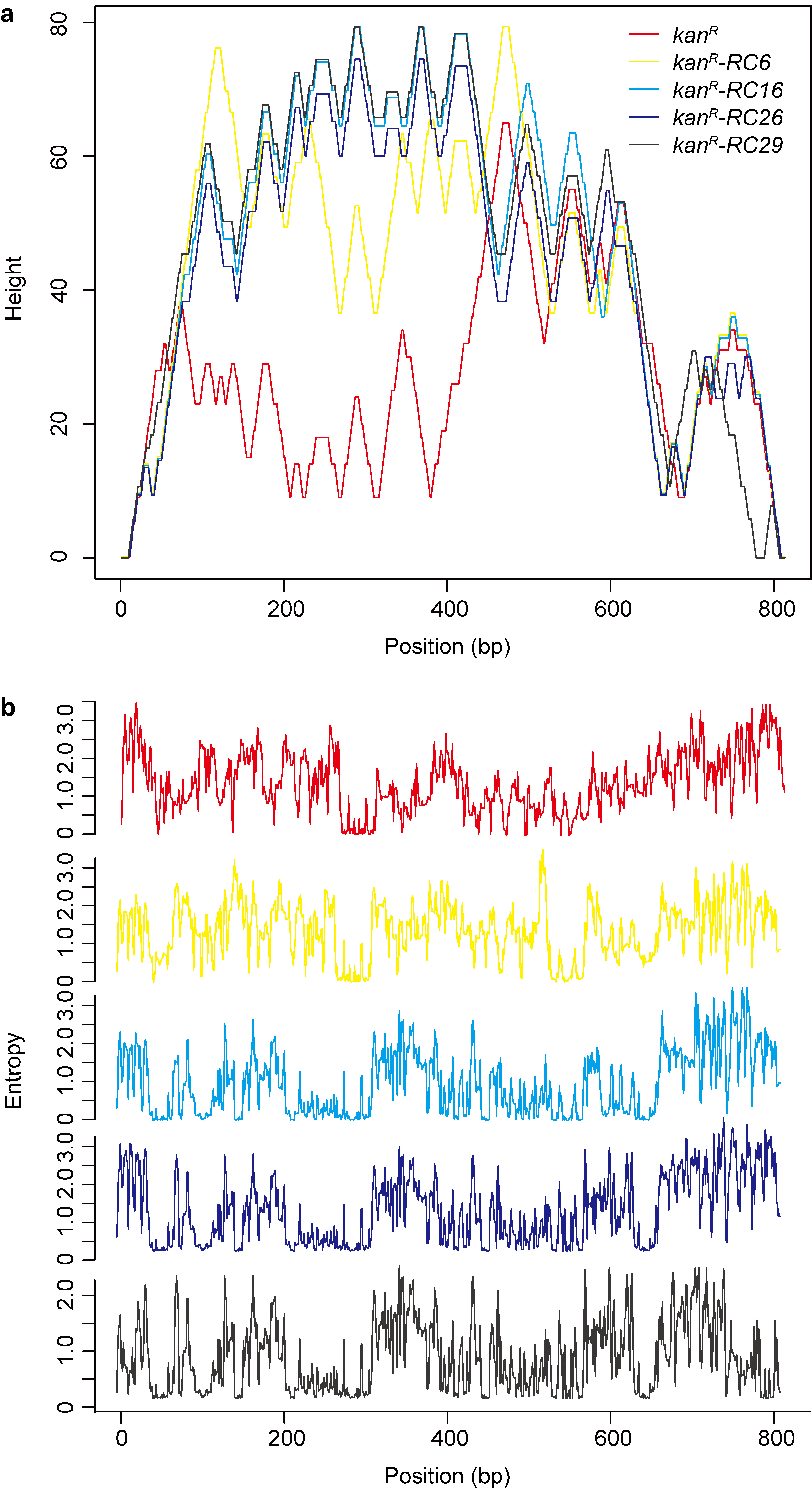


**Supplementary Figure 8. Potential secondary structures of the mRNA transcribed from the wild-type *kan^R^* and *kan^R^*-*RC*s.** The mountain plots represent minimum free energy (MFE) structure^3-5^. The heights represent the number of potential base pairs in a defined position, where plateaus, peaks and slopes represent loops, hairpin loops and helices, respectively (**a**). The positional entropy for each position is presented in (**b**).


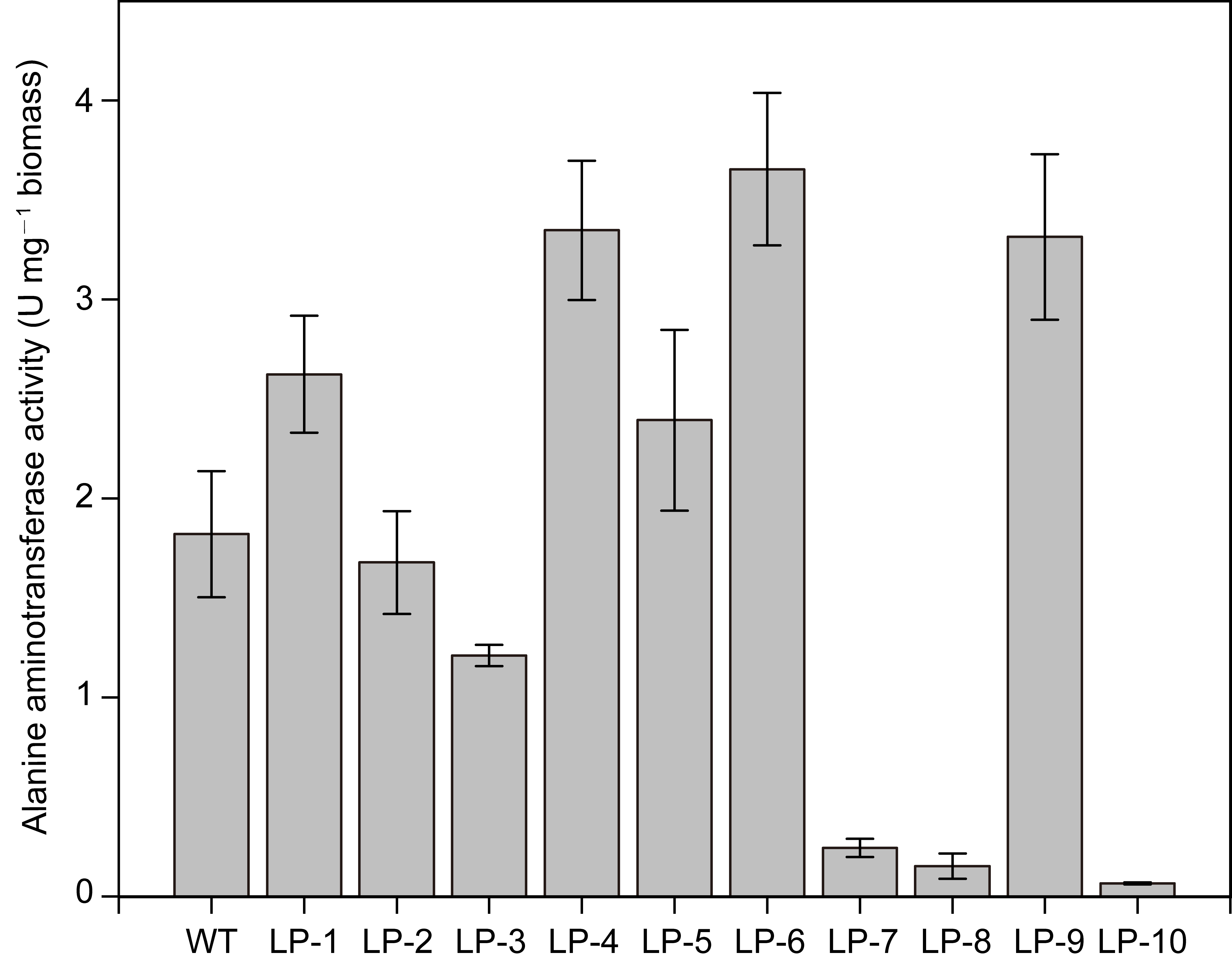


**Supplementary Figure 9. The activities of alanine aminotransferase.** Values and error bars represent the mean and s.d. (*n*=3).

**Supplementary Table 1 Candidate key mutations related to L-leucine accumulations in the selected mutants**

| Selected mutants | LP-4 | LP-7 |
| --- | --- | --- |
| *leuE* | V79I, S104N, A158T | V79I, S104N, A158T |
| *avtA* | P97A, N328D, K353E | P97A, N328D, K353E |
| *ivbL* | L6F, P15L | L6F, P15L |
| *ilvC* | I103V, G310S, G456A | I103V, G310S, G456A |

**Supplementary Table 2 The tolerances of the wild-type strain and the selected L-leucine overproducers to the L-leucine analogue L-2-aminobutyric acid**

| Strains | L-2-aminobutyric acid (g l^–1^) | | | | | | | | |
| --- | --- | --- | --- | --- | --- | --- | --- | --- | --- |
|  | 0 | 0.1 | 0.2 | 0.4 | 0.6 | 0.8 | 1.0 | 1.2 | 1.5 |
| WT | + | + | + | + | + | + | + | – | – |
| LP-4 | + | + | + | + | + | + | + | + | + |
| LP-7 | + | + | + | + | + | + | + | + | + |

"+" indicates cell growth, "–" indicates no growth.

**Supplementary References**

1. Li G-W, Oh E, Weissman JS. The anti-Shine–Dalgarno sequence drives translational pausing and codon choice in bacteria. *Nature* **484**, 538 (2012).

2. Zuker M, Stiegler P. Optimal computer folding of large RNA sequences using thermodynamics and auxiliary information. *Nucleic Acids Res* **9**, 133-148 (1981).

3. Mathews DH, *et al*. Incorporating chemical modification constraints into a dynamic programming algorithm for prediction of RNA secondary structure. *Proc Natl Acad Sci USA* **101**, 7287-7292 (2004).

4. Lorenz R, *et al*. ViennaRNA Package 2.0. *Algorithms Mol Biol* **6**, 26 (2011).

5. Gruber AR, Lorenz R, Bernhart SH, Neuböck R, Hofacker IL. The vienna RNA websuite. *Nucleic Acids Res* **36**, W70-W74 (2008).
