# Supplementary material for: Utilization of rare codon-rich markers for screening amino acid overproducers
Source: Nat Commun. 2018 Sep 6;9:3616. doi: 10.1038/s41467-018-05830-0 (PMC6127279; doi:10.1038/s41467-018-05830-0)
Supplement: Supplementary file 2 — Descriptions of Additional Supplementary Files [file 41467_2018_5830_MOESM2_ESM.pdf]

Description of Additional Supplementary Files

File Name: Supplementary Data 1
Description: Strains and plasmids used in this study.

File Name: Supplementary Data 2
Description: Sequences of the rare codon-rich genes used in this study.

File Name: Supplementary Data 3
Description: Oligonucleotides used in this study.
